# The Psychological Challenges of Emergency Medical Service Providers During Disasters: A Mini-Review February 2022

**DOI:** 10.3389/fpsyt.2022.773100

**Published:** 2022-02-28

**Authors:** Abdullah Abdulaziz Alghamdi

**Affiliations:** Department of Emergency Medical Services, Prince Sultan Bin Abdulaziz College for Emergency Medical Services, King Saud University, Riyadh, Saudi Arabia

**Keywords:** emergency medical service providers challenges, emergency medical service providers mental health, emergency medical service providers in disasters, PTSD, disasters mental health

## Abstract

The provision of emergency medical services (EMS) is an inherently stressful job. Depression, anxiety, and posttraumatic stress disorder (PTSD) are common psychological challenges affecting EMS providers. As disasters increase worldwide, the need for EMS also increases, as they are an essential part of any disaster management system. Studies have shown that EMS providers experience psychological challenges due to disaster response without receiving the needed psychological support. There is a scarcity of research focusing on EMS providers' psychological challenges in disaster times, especially in the Eastern world. This review highlights the psychological challenges faced by EMS providers in disaster times and discusses the amount of mental health care they receive. By emphasizing the need for mental health support, more research can be conducted to view EMS providers' perspectives on mental health needs before, during, and after disasters, and EMS policy makers can find programs to meet EMS providers' mental health needs, which might reflect positively on EMS and disaster management systems.

## The Psychological Challenges of Emergency Medical Service Providers During Disasters

The primary goal of disaster management is saving lives; hence, emergency medical service (EMS) providers are an essential part of any disaster management system (1). In disaster times, they are required to act quickly and efficiently to save lives and minimize injuries. Natural and human-made disasters are increasing worldwide, resulting in loss of life and property and damage to the infrastructure (2). Moreover, disasters are often associated with horrible scenes, loss of life, permanent disability, and lack of emotional support (3). A population can be psychologically impacted for years when experiencing disastrous events, such as earthquakes. For instance, in the aftermath of the 2011 L'Aquila earthquake, Bianchini et al. (4) found that almost 60% of college student survivors had different levels of depression and anxiety. Likewise, a study found that posttraumatic stress disorder (PTSD) prevalence among young people was high 3 years after experiencing a devastating earthquake (5). Hence, disasters can negatively affect a population's mental health, including that of first responders, such as police and EMS providers.

EMS providers find themselves under high pressure because they are exposed to dangers associated with the EMS occupation, such as being injured and the transmission of infection during pandemic times (e.g., COVID-19). The negative impact may extend beyond physical injuries to the psychological aspects of life. Consequently, this can affect the provision of emergency health care in routine work and disaster times (6). In recent years, there has been growing concern regarding EMS providers' mental health during routine work. Still, limited research has investigated their mental health in disaster times. This review aimed to investigate the psychological challenges that disaster response can have on EMS providers.

## Methods

### Search Strategy

PubMed, Medline, and Google Scholar databases were searched for studies published from 2011 to 2021. These databases and the time frame were determined to ensure a comprehensive search for current knowledge on the topic. The inclusion criteria were primary sources, peer-reviewed academic journals published in English, and articles that aimed to point out psychological challenges among EMS providers in disaster times. Exclusion criteria were secondary and tertiary sources, articles in a language other than English, and articles that did not mention EMS providers in a separate category. All database searches included the terms “paramedics,” “EMS,” “first responders,” “psychological challenges,” “mental health challenges,” “mental health awareness,” and “disasters.” The keywords were combined using AND and OR to access relevant articles.

The search focused on EMS providers. Studies that used general terms to describe the sample (e.g., first responders and emergency responders) were included if paramedics, ambulance personnel, or EMTs were mentioned in the sample as a separate category. One study (7) referred to EMS providers as first responders' health professionals. According to Petrie et al. (8), various phrases can describe EMS providers (e.g., emergency medical technicians, paramedics, and ambulance personnel). This variation was evident during the literature search. Therefore, the term “EMS providers” was chosen to identify emergency medical responders who provide medical services in prehospital settings.

**Figure 1:**
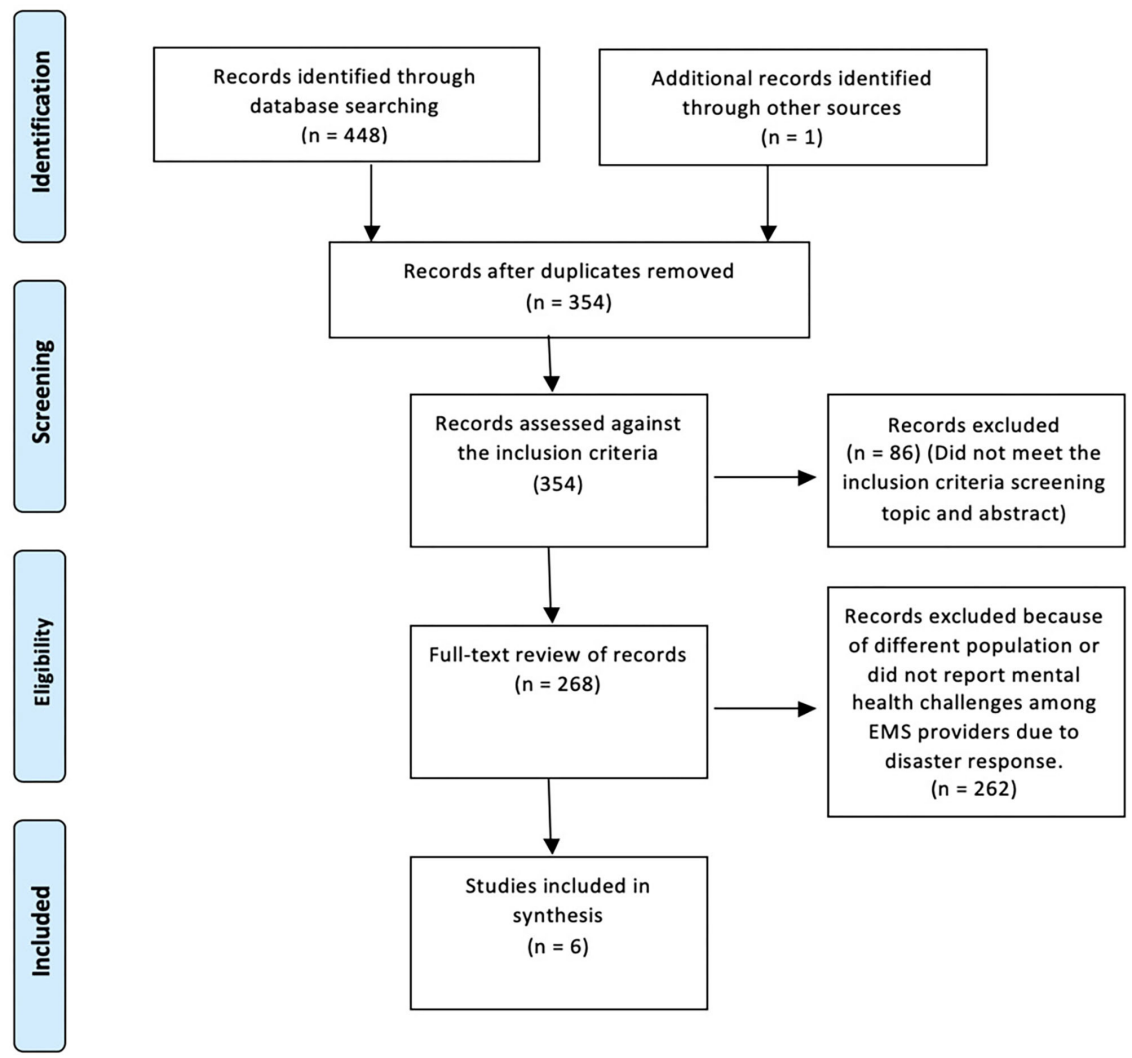
PRISMA flow chart showing the search strategy.

A total of 449 articles were identified based on database searching. Then, 354 articles were included based on title screening. From these, 268 were assessed against the inclusion criteria. Fifty-three articles were considered for full-text review. A total of six articles, five quantitative and one qualitative, were found to meet the inclusion criteria and are presented in a summary table of the review (Table 1). The PRISMA flow-chart was used to represent the included and excluded studies (13) (Figure 1).

**Table 1:** Review table.

**References**	**Study aim**	**Country**	**Study type**	**Methods**	**Sample**	**Findings and recommendations**	**Limitations**	**Theme**
Skogstad et al. (9)	To investigate the prevalence of PTSD among first responders, including EMS responders, after the 2011 terrorist attacks in Norway.	Norway	Quantitative cross-sectional study	Questionnaire	A purposive sample of 238 first responders, including 89 paramedics.All were male. Most of the participants' (60/89) ages ranged from 30 to >50 years old. 84/89 of the EMS group had >5 years of experience.	The study found very low prevalence of PTSD among all participants, including the 89 EMS providers. *Effective training and preparedness may have led to the positive psychological results*. **Recommendation:** Future research among this group to identify what made EMS providers resilient and able to overcome psychological challenges.	1. Most of the participants were men, which could have resulted in them not reporting some symptoms that could make them feel weak. 2. Absence of some emergency responders may have resulted in bias. 3. Long time between the first and second questionnaires, which may have affected the results.	Low prevalence of mental health challenges among emergency responders.
Yip et al. (10)	To investigate the mental and physical health issues among emergency responders, including EMS providers, after the response to 9/11 attack.	USA	Quantitative observational cohort study	Retrieve data from the electronic health records of FDNY Bureau of Health Services (BHS).	A purposive sample of 2,281 medical records (recorded in 2013) of EMS providers who responded to that 9/11 attacks. N = 1,795 M *N* = 486 F Age range (30–42 years). Years of experience not mentioned.	The study found that, after 12 years, some EMS providers were still having: - PTSD (*n* = 159, 7.0%) - Depression (*n* = 380, 13.7%) - Harmful alcohol use (*n* = 68, 3.0%) **Recommendation:** Continuous psychological monitoring and treatment of EMS providers.	The authors did not have access to participants' physical and mental information before 9/11 or information from databases other than FDNY-BHS.	EMS providers' vulnerability to mental health challenges. EMS providers' vulnerability to long-term health challenges. The need for mental health awareness programs.
Hsiao et al. (11)	To identify the prognostic factors that could have led to PTSD among EMTs who responded to the 2016 Taiwan Earthquake.	Taiwan	Quantitative longitudinal study	Two questionnaires were distributed 1 month and then 6 months after the disaster.	Voluntary sampling of 38 EMTs. All were male. Median age (35 years). Median years of experience (13.5 years).	Psychological challenges can develop immediately after the disaster response and remain high for 6 months and more. The total scores showed that *N* = 19/38 reported PTSD after one month and *N* = 13/38 reported PTSD symptoms after 6 months. **Recommendation:** Psychological challenges should be clarified before disaster response. Short- and long- term counseling stress management programs.	1. The sample size was small and consisted of only men. 2. Using a self-report questionnaire rather than a clinician's diagnosis could have affected the value of the study. 3. No information showed whether the EMTs had other missions during the 6 months.	EMS providers' vulnerability to mental health challenges. The need for mental health awareness programs.
Smith and Burkle (12)	To get EMTs' and paramedics' reflections on the long-term impact of mental health challenges after response to the 9/11 attacks.	USA	Qualitative phenomenological	Face-to-face interviews	Purposive & snowball sampling of 54 EMS providers. *N* = 18 paramedics. *N* = 36 EMTs. *N* = 42 M *N* = 12 F Age range (39–68 years). Years of experience not mentioned.	Most of the participants reported long-term mental health challenges, such as guilty feelings, anxiety, and nightmares due to responding to the 9/11 attacks. *N* = 24 of the participants were not provided with psychological support by their employers. *N* = 43 reported PTSD symptoms (50% did not seek support). *N* = 8 reported anxiety. **Recommendation:** Continuous psychological monitoring of EMS providers after disasters.	1. Influence of assumptions. 2. Sampling bias due to the small sample number. 3. Lack of clinician's diagnosis of participants.	EMS providers' vulnerability to long-term health challenges. The need for mental health awareness programs.
Motreff et al. (7)	To assess the long-term impact of PTSD among first responders, including EMS providers after Paris 2015 attack.	France	Quantitative	Questionnaire	Purposive sampling of 614 first responders, including 230 EMS providers (mentioned as first responders' health professionals). *N* = 148 M *N* = 82 F Mean age (43 years). Years of experience not mentioned.	Between 8 months and 1 year after responding to the 2015 Paris attack, *n* = 10/230 (4.4%) had PTSD symptoms, *n* = 24/230 (10.4) had partial PTSD symptoms. Lack of psychological awareness was associated with PTSD symptoms. **Recommendation:** Systematic education about the potential mental health challenges associated with disaster response.	Small sample number.	EMS providers' vulnerability to long-term health challenges. The need for mental health awareness programs.
Yacout et al. (1)	To investigate levels of PTSD and burnout among paramedics following COVID-19.	Egypt	Quantitative descriptive exploratory	Questionnaire	Purposive sampling of 68 paramedics. All were male. Mean age (40 years). Most of the participants (69%) had work experience >10 years.	*n* = 23/68 (33.8%) had emotional exhaustion, and n = 19/68 (27.9) had high levels of emotional exhaustion. Participants reported different degrees of burnout syndrome and PTSD. **Recommendation:** Continuous screening of paramedics to evaluate psychological challenges associated with the EMS profession Mental health and stress management awareness programs are needed. A plan to support paramedics' mental health.	Not reported	The need for mental health awareness programs.

Three themes were recognized: disaster response leads to long-term psychological challenges, psychological preparedness can mitigate the effect of psychological stress, and years of experience have little correlation with severity of psychological challenges.

### Findings

Yacout et al. (1) investigated the levels of emotional exhaustion, fatigue, and PTSD levels among 68 EMS providers due to work during the COVID-19 pandemic. According to the results, 23 (33.8%) had emotional exhaustion, and 19 (27.9%) reported high levels of emotional exhaustion. The participants also showed different degrees of PTSD and burnout associated with demographic variables, such as age, work experience, educational level, and self-care of mental and physical health. Routine mental health screening and mental health awareness programs can help EMS providers overcome mental health conditions (1).

### Disaster Response Leads to Long-Term Psychological Challenges

A correlation exists between working in disaster times and long-term psychological challenges. For instance, a longitudinal study conducted after the 2017 Taiwan earthquake among 37 EMS providers showed that 19 EMS providers (51.3%) reported PTSD symptoms after 1 month, with 13 of those reporting PTSD symptoms at a 6-month follow-up (11). In their study, the authors suggest that mental health challenges associated with disaster response may develop immediately after disasters and continue for months or even years if not treated. The study did not mention whether any of the paramedics received psychological support.

Moreover, responding to the 2001 9/11 attack in the United States was one of the most traumatic events that severely affected thousands of first responders' wellbeing in the long term. The attack has directed attention toward the long-term mental health challenges among first responders, including EMS providers. Smith and Burkle (12) found that a group of EMS providers were still experiencing feelings of guilt, anxiety, and nightmares 15 years after responding to the 9/11 attack. For instance, 43 out of 54 EMS providers (79.6%) who responded to the 9/11 attacks reported PTSD, and eight reported anxiety symptoms. Nonetheless, 50% of the participants stated that their EMS organization did not offer them psychological support (12).

Likewise, Yip et al. (10) conducted an observational study 12 years after the 9/11 attack, consisting of 2,281 EMS responders who responded. They found that 159 (7.0%) of the participants had PTSD and 380 (16.6%) had depression. The significance of these results include that the participants represent a wide range of paramedics who have suffered from chronic stress for an entire decade. Thus, it can be said that EMS providers are subjected to long-term psychological burdens due to disaster response, which require interventions before, during, and after disasters.

### Psychological Preparedness Can Mitigate the Effect of Psychological Stress

Enlightening EMS providers to what they should expect to deal with mentally and physically during routine work and disaster times can help them overcome potential psychological challenges. Effective preparedness and organizational support can prevent or decrease psychological challenges associated with the EMS profession before, during, and after disasters. For example, Skogstad et al. (9) conducted a study among 238 first responders, including 89 EMS providers, to investigate the prevalence of PTSD in the aftermath of the 2011 terrorist attacks in Norway. Even though the attack exposed first responders to many dead bodies and victims, the results showed a low percentage (<2%) of PTSD symptoms among them. The results showed that only one EMS provider showed possible symptoms of PTSD. However, the study found that most EMS providers, 80/89 (90%), participated in disaster drills before the attacks. This conclusion suggests that good training, preparedness, and peer support may have led to low psychological burden levels in the aftermath of the disaster among all first responders.

In contrast, a lack of mental health awareness can lead to psychological challenges after responding to major incidents. For instance, a study conducted 1 year after the 2015 terror attacks in France with 230 EMS providers showed that 34 EMS providers (14.8%) had PTSD and partial PTSD symptoms (7). More importantly, the study suggests that the lack of awareness about the potential psychological challenges associated with response to major emergencies was associated with PTSD symptoms. The authors recommended establishing awareness and training programs regarding the potential mental health challenges related to traumatic events.

### Years of Experience Have Little Correlation With Severity of Psychological Challenges

The number of years of experience reported for EMS providers in the review ranged from 5 to 14 years for fieldwork. Hence, it is very likely that even the most experienced EMS provider (14 years) may experience psychological challenges due to working in disaster times, just like those with less experience (5 years). The results can indicate that EMS providers, regardless of their years of work experience in the EMS field, need to receive the proper intervention to deal with EMS work-related mental health challenges before fieldwork.

## Discussion

The purpose of this paper was to provide an up-to-date review of the current knowledge about the psychological challenges that EMS providers face during disaster times. The results indicate that the psychological challenges associated with disaster response can develop into chronic mental illness, negatively impacting their work efficiency and threatening the safety of EMS providers and their patients. A study reported that experiencing disasters, such as deadly earthquakes has a high possibility of developing PTSD among other psychological challenges (14). PTSD symptoms have been correlated with lower performance and impaired decision-making (15). In addition, according to Leykin et al. (16), depression symptoms can significantly impact problem-solving abilities and decision-making. Such mental health symptoms can result in ineffective performance of EMS providers, especially during disasters, as they work with multiple patients in a hectic work environment. Moreover, due to the significant risk that EMS providers face in disaster times, groups of EMS providers and community members have called for setting limitations on EMS providers' duties to treat patients during disasters and suggested limitations on EMS providers who have experienced psychological challenges (17).

Most of the studies included in the review investigated the long-term psychological impacts among EMS providers (7, 10–12). Only one study was conducted regarding the response to a large-scale emergency—the COVID-19 pandemic (1). In addition to examining the long-term-psychological challenges, conducting studies that investigate EMS providers' mental health challenges during times of disaster or shortly after its occurrence can lead to quickly recognizing psychological challenges and offering the proper assistance to EMS providers before they become chronic illnesses.

Moreover, the majority of the included studies were carried out in Western countries (United States, Norway, French). Only one recent study was conducted in the Middle East (1). The literature review revealed a scarcity of research focusing on mental health challenges among EMS providers in Asia and the Middle East. Also, the majority of the participants in all included studies were male, which does not allow researchers and policy makers to examine the differences between genders and make the appropriate recommendations that suit each gender.

Additionally, not enough qualitative studies were conducted to allow the participants to express their feelings regarding work stressors associated with the EMS profession. However, due to the small amount of literature and the focus on the Western side of the world, the results cannot be generalized, and more studies should be conducted in the Middle East and the Eastern world to provide a more in-depth understanding of their mental health awareness and experiences in overcoming psychological challenges before, during, and after disasters.

The included studies show that all levels of work field experience appear to be equally affected by psychological challenges, indicating that even EMS providers who have long experience in the field may develop psychological challenges such as anxiety, depression, and PTSD. In a statistic published by the Health and Safety Executive (18), it was evident that male employees aged from16 years to over 55 years had almost similar levels of work-related stressors (e.g., stress, depression, anxiety). No studies were found investigating years of experience and psychological challenges among EMS providers as a focused group. However, as the third theme suggests, the association between the nature of the EMS profession and psychological challenges among different age groups calls for ongoing mental health screening and intervention. Further research is needed to investigate the correlation between years of experience and levels of psychological challenges among EMS personnel.

As most studies among EMS providers have been conducted long after the response to disasters, there is little information regarding their mental health awareness and their coping mechanisms from the response time until the time of the study. Such information can help in recognizing the appropriate short, medium, and long term measures for reducing psychological damage and providing guidance and counseling to strengthen EMS providers' sense of coherence. A sense of coherence mirrors how people view their lives in terms of mental health and the coping mechanisms they could benefit from to overcome mental illness (19). Behnke et al. (19) found that EMS providers who had a stronger sense of coherence were more resilient to mental health challenges than those who had a weaker sense of coherence. EMS agencies should adopt practical solutions in order to ease the psychological challenges among their employees, such as awareness programs, booklets, and therapeutic intervention (e.g., cognitive behavioral therapy).

EMS providers may benefit from using the technology to receive self-help psychological support. Indeed, A study among young adults with anxiety disorders concluded that both parson-to-person cognitive behavioral therapy and computerized cognitive behavioral therapy (cCBT) can improve anxious symptoms, and enhance social life (20). The authors of the study concluded that cCBT can facilitate access to mental health support and have the same outcome of person-to-person cognitive behavioral therapy. Therefore, such intervention may help to mitigate short, medium, and long term mental health challenges among EMS providers because of its possible effectiveness, and the easy access to the service, which may encourage those who work for long shifts to utilize it.

### Limitations and Strengths

The included studies reported two common limitations: small sample sizes and the possibility of bias. Such limitations might have affected the findings and a lack of generalizability. In addition, some limitations were identified during the literature review. For example, none of the included studies provided practical solutions for easing psychological challenges.

It is important to acknowledge that this review has some limitations. First, it included studies within only the last 10 years (2011–2021). Studies before 2011 may have additional insights that could change the conclusion. Second, the review included a limited number of studies; most reported a small sample number, which might have resulted in bias. However, a strength of this review was that EMS providers were the focus group or were mentioned in a separate category in all included studies, which helped identify the psychological challenges from EMS providers' viewpoint.

## Conclusion

EMS is considered a keystone of any disaster management system, and EMS providers play a significant role in saving lives during disasters. Their work is mentally demanding and requires complete focus and accurate actions to save lives. This review revealed that psychological challenges among EMS providers increase due to exposure to traumatic events in disaster times, which could negatively impact their work efficiency (12). In addition to physical harm, the review suggests that emotional stress could also be considered one of the dangers associated with disaster response among EMS providers. Without proper mental health support, the disaster management system could be affected when the need for emergency medical care increases. One of the review's trends is that psychological preparation for EMS providers is just as necessary as physical preparation for disaster response.

According to the World Health Organization (21), psychological support programs at work can effectively promote mental health. Therefore, establishing mental health support units at EMS agencies and awareness efforts, such as providing booklets, awareness programs, and offering cognitive behavioral therapy, either person-to-person or *via* technology, can help increase mental health awareness and mitigate psychological challenges among EMS providers. Overall, more research and mental health awareness/support are needed to ensure a healthy EMS system and disaster management system. Mental health support services should be available to EMS providers before, during, and after disasters.

## Author Contributions

AA as the main and only researcher, has completed the work individually with no assistance. Author contributed to the article and approved the submitted version.

## Conflict of Interest

The author declares that the research was conducted in the absence of any commercial or financial relationships that could be construed as a potential conflict of interest.

## Publisher's Note

All claims expressed in this article are solely those of the authors and do not necessarily represent those of their affiliated organizations, or those of the publisher, the editors and the reviewers. Any product that may be evaluated in this article, or claim that may be made by its manufacturer, is not guaranteed or endorsed by the publisher.
